# Effect of voice therapy with or without transcutaneous electrical stimulation on recovery of injured macroscopically intact recurrent laryngeal nerve after thyroid surgery

**DOI:** 10.1007/s00405-020-05806-1

**Published:** 2020-01-24

**Authors:** Martin Formánek, Radana Walderová, Šárka Baníková, Irina Chmelová, Debora Formánková, Karol Zeleník, Pavel Komínek

**Affiliations:** 1 Department of Otorhinolaryngology and Head and Neck Surgery, University Hospital Ostrava, 17. Listopadu 1790, 70852 Ostrava, Czech Republic; 2 Department of Craniofacial Surgery, Faculty of Medicine, University of Ostrava, Syllabova 19, 703 00 Ostrava, Czech Republic; 3 Department of Rehabilitation and Physical Medicine, University Hospital Ostrava, 17. Listopadu 1790, 70852 Ostrava, Czech Republic

**Keywords:** Electrical stimulation, Transcutaneous electrical nerve stimulation, Recurrent laryngeal nerve, Vocal cord paralysis, Voice training, Treatment outcome

## Abstract

**Purpose:**

Electrical stimulation-supported therapy is an often used modality. However, it still belongs to experimental methods in the human larynx. Data are lacking with which to evaluate the real effect in recurrent laryngeal nerve injury. The aim of this study was to investigate whether transcutaneous electrical stimulation added to voice therapy has a beneficial effect compared to voice therapy alone on vocal fold movement recovery in the case of an injured macroscopically intact recurrent laryngeal nerve.

**Methods:**

Adults with unilateral vocal fold paralysis after thyroidectomy, in which the recurrent laryngeal nerve was left macroscopically intact, were included in this case–control study performed in tertiary referral hospital between September 2006 and June 2018. Among 175 eligible participants, 158 were included. Compliance with 6 months follow-up was 94.3%. Interventions: medicament therapy and voice therapy (group 1) vs. medicament therapy and voice therapy and transcutaneous electrical stimulation (group 2). Main outcome: vocal fold movement.

**Results:**

A total of 149 patients were included in the analysis (group 1, 89 patients; group 2, 60 patients). The groups were homogenous. In groups 1 and 2, 64% and 60% of vocal folds, respectively, were improved after 6 months (*P* = 0.617). No difference was found between patients who improved and patients who did not improve.

**Conclusions:**

Adding transcutaneous electrical stimulation to voice therapy provided no beneficial effect on the recovery of vocal fold movement. Therefore, its indications should be re-evaluated; it is questionable whether stimulation should be routinely recommended.

## Introduction

Recurrent laryngeal nerve (RLN) lesions cause vocal fold paralysis, and the symptoms are dependent on the position in which the vocal fold remains [1]. The lesions can be almost asymptomatic to unprofessional hearing in as many as 30% of patients or can cause incomplete glottic closure, which can lead to dysphonia, dysphagia, or life threatening aspiration in some cases [2]. These symptoms can significantly deteriorate the patient´s quality of life.

RLN lesions are most often iatrogenic, caused by operations on the thyroid or parathyroid gland. Thyroid gland surgery is becoming more and more frequent, with hundreds of thousands of worldwide surgeries performed each year [3, 4]. RLN injury is one of its most feared complications, with rates of permanent damage from 0.5 to 3% of nerves at risk and several times more frequent transient damage [5–9]. The number of injured RLNs is likely even higher because not every patient routinely undergoes a vocal fold check after surgery, such as when surgery is performed by general surgeons [9]. The main principal of safe surgery is exposure and gentle preparation near the RLN [10]. However, the RLN is very sensitive. Even a macroscopically intact nerve can be harmed through a variety of mechanisms, including stretching, crushing, thermal, or electrical injury [11]. Neurapraxia or axonotmesis can be present in such a case. Only temporary loss of function occurs in neurapraxia and, dissimilar to axonotmesis, Wallerian degeneration does not occur. However, neurapraxia and axonotmesis cannot be differentiated macroscopically [12].

The main goal of post-operative care is to reduce edema, facilitate nerve regeneration as much as possible, and reduce the number of cases of permanent paralysis. Voice therapy is currently recommended in the case of postoperative vocal fold paralysis [13, 14]. However, success is far from certain, and whether therapy has an effect on the recovery of vocal fold motility or only provides compensation until regeneration is complete is unclear [13, 14]. Therefore, other procedures facilitating regeneration are being investigated, including electrical stimulation.

Electrical stimulation-supported therapy is a well-known modality in the field of orthopedics and physical medicine [15]. However, it is still an experimental method in the human larynx. Data are lacking for the evaluation of its real effects in the case of RLN injury. The main aim of our prospective case control trial was to investigate whether transcutaneous electrical stimulation added to voice therapy has a beneficial effect compared to voice therapy on the recovery of an injured macroscopically intact RLN after thyroid or parathyroid surgery.

## Materials and methods

This case–control study was approved by the Ethics Committee under the identifier 1117 and performed in accordance with the Declaration of Helsinki and applicable regulatory requirements using good clinical practice. Written informed consent was obtained from the patients before initiating any procedure. The study was performed between September 2006 and June 2018 in a tertiary referral hospital. Because the study began in 2006, the study was not registered in a public trials registry. All authors reviewed and approved the final manuscript.

### Participants

Adults with unilateral vocal fold paralysis after thyroidectomy or parathyroidectomy during which the RLN was left macroscopically intact were included in the study. Patients were divided into two groups according to whether they underwent additional transcutaneous electrical stimulation during their postoperative rehabilitation. All patients (both groups) were treated with corticosteroids, B vitamins, and voice therapy. Patients in group 2 were also treated with added transcutaneous electrical stimulation. Exclusion criteria were vocal fold paralysis due to loss of RLN continuity, previous voice or electrical stimulation therapy, presence of other conditions that could possibly interfere with the therapy, such as cognitive deficits or other serious diseases, or refusal to sign informed consent. Among 175 eligible participants, 158 were included. Added transcutaneous electrical stimulation was offered to all not contraindicated patients and patients were given a free choice of therapy. Patients who did not want electrical stimulation or in whom the procedure was contraindicated were assigned to group 1. Patients treated with added transcutaneous electrical stimulation were assigned to group 2.

### Data acquisition and follow-up

Vocal fold mobility was evaluated using a 2.7-mm flexible HD endoscope (Olympus Europa SE & Co., Hamburg, Germany) 1 day and 6 months after the surgery. Vocal fold movement was scored as none (= not improved), normal (= improved), or partial (= partially improved) if moving vocal fold did not reach the midline.

### Medicament therapy

Medicament therapy started 1 day after the surgery. All patients were treated with intravenous methylprednisolone sodium succinate at a decreasing dose for 7 days (250 mg for 3 days, 125 mg for 2 days, 80 mg for 2 days), and then with 20 mg methylprednisolone orally for 3 days. All patients were also treated with 80 mg benfotiamine, 180 mg pyridoxine hydrochloride, and 0.5 mg cyanocobalamin orally every 8 h for 30 days.

### Voice therapy

Voice therapy sessions started 3 days after surgery. Exercise for glottis closure correction was applied. The patient was instructed to pronounce with pushing syllables “ha”, “he”, “hy”, “ho”, “hu”, which was accompanied by thoraco-petal upper limbs flexing. Patient was educated by a phoniatrician and, from the second session, performed under the guidance of a speech therapist. A total of five sessions were held with 7–10 days interval to adequately educate the patient. The patient was instructed to perform exercises at home every day for at least 15 min for 6 months.

### Transcutaneous electrical stimulation

**Fig. 1:**
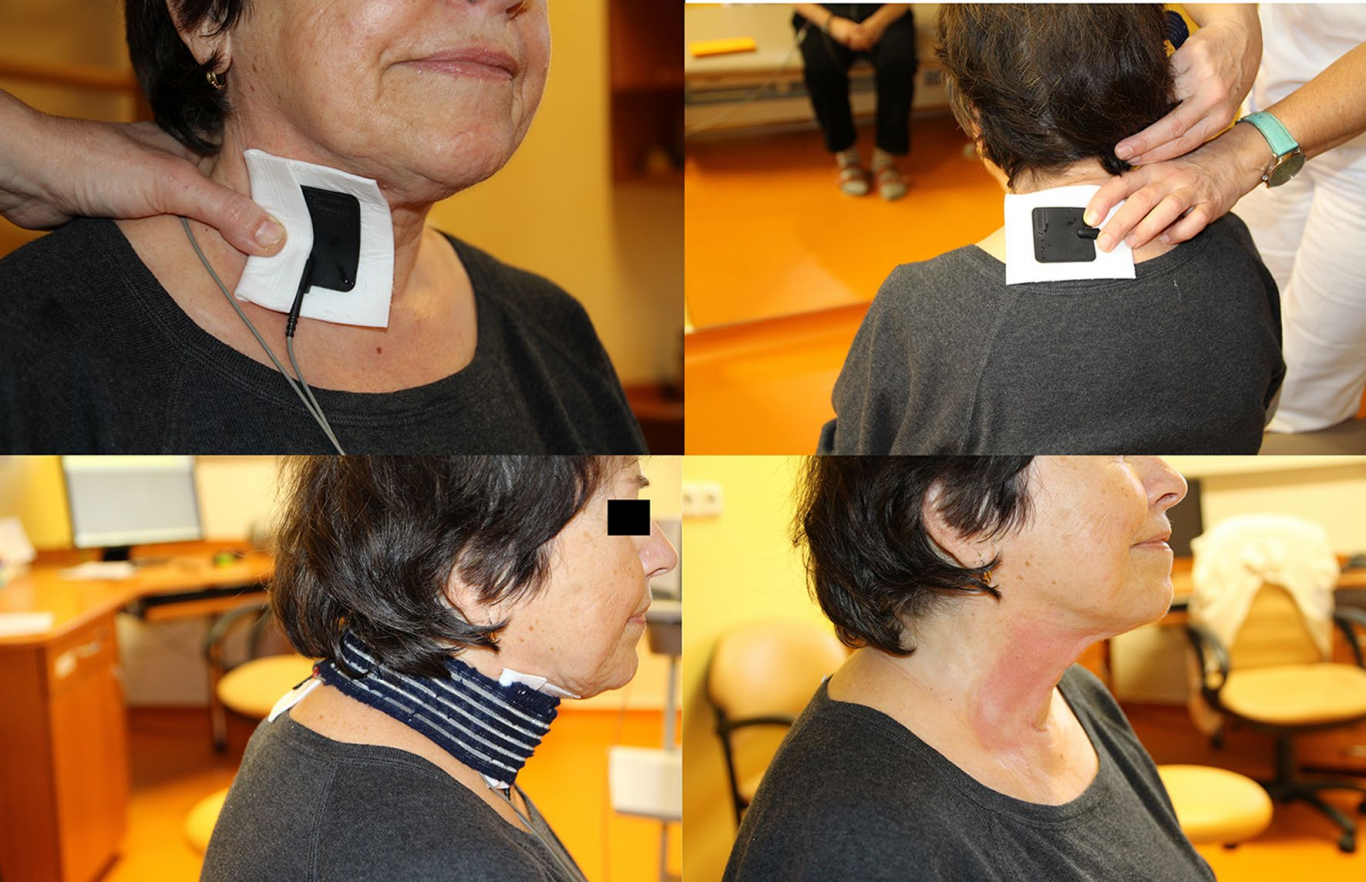
Transcutaneous electrical stimulation—electrodes placement in case of right vocal fold paralysis

Electrical stimulator Gymna (Gymna NV, Bilzen, Belgium) was used for stimulation. The stimulation was performed with the sitting patient in the outpatient department by an experienced physiatrist educated in voice therapy. The cathode was placed at a muscle motor point on the skin above the thyroid cartilage unilateral to the vocal fold paralysis, and the anode was placed on the skin above the contralateral trapezius muscle (Fig. 1). Direct current was applied for 10 min (oblique impulses with slow growing intensity, pulse width 500 ms, frequency 0.4 Hz, intensity corresponding to individual motor threshold). The patient underwent passive stimulation with maximum effort to achieve the peak of the pulse in the first 2 min. This was facilitated by the audio signal from the stimulator. The patient accompanied each impulse with upper limb flexing and pronounced with pushing gradually prolonged syllables “ha”, “he”, “hy”, “ho”, “hu” over 8 min (active stimulation). The intervention was performed three or four times a week for a total of 20 interventions at a maximum interval of two business days. Contraindication to stimulation was pacemaker, cochlear implant, metal implants in the head and neck region, severe arterial hypertension or hypotension, complicated wound healing, neck skin pathology, fever, or psychological disorders.

### Statistical analysis

Descriptive statistics, such as the arithmetic mean, standard deviation, and absolute and relative frequency tables, were used for data processing. Pearson’s Chi-squared test and two-sample *t* test with equal variances were used for comparisons between groups. The statistical tests were assessed using a significance level of 5%. Therapy was considered successful if there was a return of vocal fold movement. The statistical analyses were performed using Stata 13 software (Stata Corp., College Station, TX, USA).

## Results

A total of 158 patients met the inclusion criteria and were included in the study. Nine patients (three in group 1 and six in group 2) were lost to follow-up (Fig. 1). The compliance with follow-up was 94.3%. A total of 149 patients with vocal fold paralysis (84 left-sided and 65 right-sided) were included in the analysis. Group 1 included 89 patients and group 2 (with electrical stimulation) included 60 patients. No differences were found between the groups in regards to average age, sex distribution, and prevalence of glandular malignant tumors (Table 1).

**Table 1:** Characteristics of the study participants

Characteristic	Group 1 (*n* = 89)	Group 2 (*n* = 60)	*P* value^a^
Age, years	55.3 (14.45)	50.5 (14.79)	0.051
Male	7 (7.9)	6 (10)	0.651
Female	82 (92.1)	54 (90)	0.651
Malignant tumor	9 (10.1)	7 (11.7)	0.764

Data are presented as mean (SD) or *n* (%)

^a^Pearson’s Chi-squared test

There were no partially improved patients. No significant difference was found between the two groups in the post-treatment improvement in vocal fold movement after 6 months (Table 2). No difference was found when patients who improved and patients who did not improved were analyzed in regards to age, sex distribution, and malignancy (Table 3).

**Table 2:** Comparison of improvement between groups

	Group 1	Group 2
Not improved	32 (36)	24 (40)
Improved	57 (64)	36 (60)
*P* value^a^	0.617	

Data are presented as *n* (%)

^a^Pearson’s Chi-squared test

**Table 3:** Comparison of improved and not improve

	Not improved (*n* = 56)	Improved (*n* = 93)	*P* value
Age, years	53.3 (16.63)	53.3 (13.55)	0.994^a^
Male	7 (53.8)	6 (46.2)	0.205^b^
Female	49 (36)	87 (64)	0.205^b^
Malignant tumor	5 (31.2)	11 (68.8)	0.764^b^

Data are presented as mean (SD) or *n* (%)

^a^Two-sample *t* test with equal variances

^b^Pearson’s Chi-squared test

## Discussion

Electrical stimulation–supported therapy is a well-known modality in the field of orthopedics and physical medicine, but is still an experimental method in the human larynx [15]. Satisfactory data on its real effect in the case of RLN injury are missing. Most data on the possible effectiveness of stimulation on RLN regeneration come from animal studies [16–18]. Ptok et al. was one of the few groups to clinically investigate this issue in humans [19]. In this prospective study, voice exercise was compared to electrical stimulation–supported voice exercises in 88 patients with unilateral vocal fold paralysis, and added electrical stimulation was superior in terms of vocal fold vibration irregularity after 3 months. This seems to be inconsistent with the results of our clinical trial. However, vocal fold movement was not really evaluated in Ptok et al.’s study, and there was no difference in maximum phonation time, which indirectly indicates how well the vocal folds are approximating/moving. Therefore, if the results are analyzed in more detail, the previous study actually supports the results of our study. Furthermore, patients with many different etiologies of paralysis (e.g., postoperative, idiopathic, post-infectious) were included in Ptok et al.’s study. In addition, patients with paralysis lasting from 2 weeks to 6 months were included, which also could have affected the results considering short follow-up and relatively slow nerve regeneration, which can take up to 6–12 months depending on the mechanism and localization of damage [9, 19].

Therefore, several issues had to be considered in order to provide valid data. Only patients with the same etiology and same duration of paralysis were included in our study in order to eliminate potential bias. A follow-up of 6 months was established in our trial because this time should be sufficient for nerve regeneration if we take into account that the RLN was injured no more than several centimeters before the entry point to the larynx. End-organ (muscle) stimulation was used, which should not accelerate nerve regeneration, but rather facilitate it [20]. Therefore, more frequent checks were not indicated and time to recovery was not monitored. Voice parameters were also not monitored, as they are dependent on the patient’s effort and not quite comparable among patients due to the paralytic vocal fold remaining in a different position each time. On the other hand, vocal fold movement is a clearly defined, easily comparable, objective parameter.

We decided not to use questionnaires for treatment co-evaluation either because of several reasons. First, they are not always accurate and reliable. Second, patient´s voice is not routinely scored before every thyroid surgery; therefore preoperative data would be missing. We could only score patient’s voice a few days after the surgery, when vocal folds are still harmed by intubation tube, which is moreover introduced for a different period of time in every patient (different duration of operation, hospitalization in an intensive care unit, etc.). Patients also recovered from major surgery. Third, we wanted to eliminate potential risk of patients being influenced by factors other than true voice satisfaction, as only follow-up after 6 months was performed and therefore only one post-treatment result was available. Questionnaire’s score could be strongly influenced by patient’s actual mood or social change, as some patients were diagnosed with thyroid malignancies and some patients were treated with mood-altering drugs.

Our trial included the largest reported number of participants. In general, the recovery rate was quite high in both groups, reaching up to 65%. This is in line with the study by Mottioli et al. in which 74 patients underwent vocal exercises, with 68.9% recovering vocal fold motility during 7 years of follow-up [13]. In contrast with other peripheral nerve treatments, no effect was found of added electrical stimulation on RLN regeneration. Even when improved and not improved patients were analyzed regards to age, sex distribution and malignancy, which could potentially influence outcome. This could be explained by the fact, that RLN is one of the most difficult peripheral nerves with which to achieve functional regeneration, especially when it is severed, as indicated by the results of end-to-end anastomoses of the transected nerve. Although immediate anastomosis of the RLN nerve is performed, it does not usually result in the functional recovery of vocal fold mobility [21].

Although only RLNs injured during thyroid or parathyroid gland surgery were included, our results are applicable to all iatrogenic injuries during which the RLN remains macroscopically intact. The follow-up should be modified according to the localization of the nerve injury.

A limitation of our study is the absence of randomization. Partial randomization was secured against by giving patients for whom stimulation was not contraindicated a free choice of therapy. This grouping slightly favored group 2 because more motivated patients could be expected in this group. Yet, the results were not better in group 2. In addition, there were almost significantly younger patients in group 2, in whom better healing could be generally expected. Lower age could also be one of the reasons for choosing to undergo stimulation.

## Conclusions

Adding transcutaneous electrical stimulation to voice therapy provided no benefit to the recovery of vocal fold movement in injured macroscopically intact RLNs after thyroid or parathyroid surgery compared to voice therapy only. Therefore, indications for electrical stimulation should be re-evaluated, and it is questionable whether stimulation should be routinely recommended considering the time demands for both the patient and the physiotherapist.
